# Investigation of the Effect of Molecules Containing Sulfonamide Moiety Adsorbed on the FAPbI_3_ Perovskite Surface: A First-Principles Study

**DOI:** 10.3390/molecules30112463

**Published:** 2025-06-04

**Authors:** Shiyan Yang, Yu Zhuang, Youbo Dou, Jianjun Wang, Hongwen Zhang, Wenjing Lu, Qiuli Zhang, Xihua Zhang, Yuan Wu, Xianfeng Jiang

**Affiliations:** School of Energy and Environment Science, Yunnan Normal University, Kunming 650500, China; 18184845393@163.com (S.Y.); ybdou@outlook.com (Y.D.); wjianjun622@gmail.com (J.W.); 18287442863@163.com (H.Z.); 15887460619@163.com (W.L.); 15087540821@163.com (Q.Z.); 15911967478@163.com (X.Z.); 15198713255@163.com (Y.W.); 17761402464@163.com (X.J.)

**Keywords:** FAPbI_3_ perovskite, surface adsorption, optoelectronic properties, first-principles

## Abstract

First-principles calculations were conducted to examine the impact of three sulfonamide-containing molecules (H_4_N_2_O_2_S, CH_8_N_4_O_3_S, and C_2_H_2_N_6_O_4_S) adsorbed on the FAPbI_3_(001) perovskite surface, aiming to establish a significant positive correlation between the molecular structures and their regulatory effects on the perovskite surface. A systematic comparison was conducted to evaluate the adsorption stability of the three molecules on the two distinct surface terminations. The results show that all three molecules exhibit strong adsorption on the FAPbI_3_(001) surface, with C_2_H_12_N_6_O_4_S demonstrating the most favorable binding stability due to its extended frameworks and multiple electron-donating/withdrawing groups. Simpler molecules lacking carbon skeletons exhibit weaker adsorption and less dependence on surface termination. Ab initio molecular dynamics simulations (AIMD) further corroborated the thermal stability of the stable adsorption configurations at elevated temperatures. Electronic structure analysis reveals that molecular adsorption significantly reconstructs the density of states (DOS) on the PbI_2_-terminated surface, inducing shifts in band-edge states and enhancing energy-level coupling between molecular orbitals and surface states. In contrast, the FAI-terminated surface shows weaker interactions. Charge density difference (CDD) analysis indicates that the molecules form multiple coordination bonds (e.g., Pb–O, Pb–S, and Pb–N) with uncoordinated Pb atoms, facilitated by –SO_2_–NH_2_ groups. Bader charge and work function analyses indicate that the PbI_2_-terminated surface exhibits more pronounced electronic coupling and interfacial charge transfer. The C_2_H_12_N_6_O_4_S adsorption system demonstrates the most substantial reduction in work function. Optical property calculations show a distinct red-shift in the absorption edge along both the XX and YY directions for all adsorption systems, accompanied by enhanced absorption intensity and broadened spectral range. These findings suggest that sulfonamide-containing molecules, particularly C_2_H_12_N_6_O_4_S with extended carbon skeletons, can effectively stabilize the perovskite interface, optimize charge transport pathways, and enhance light-harvesting performance.

## 1. Introduction

In recent years, organic–inorganic hybrid perovskite solar cells (PSCs) have quickly become one of the most promising photovoltaic technologies [1]. In just a few years, the power conversion efficiency (PCE) of PSCs has improved significantly, from 3.8% [2] to 27% [3]. However, non-radiative carrier recombination remains a key factor limiting the open-circuit voltage (Voc), indicating that there is significant room for improvement [4]. Presently, the efficiency of PSCs has yet to reach the theoretical Shockley–Queisser limit of 33.7% [5]. Among commonly used light-absorbing materials of PSCs, MAPbI3 and FAPbI3 have garnered considerable attention. FAPbI3, in particular, has garnered significant research due to its narrower bandgap (1.48 eV) and more symmetrical crystal structure [6], both of which are advantageous for enhanced near-infrared absorption [7], improved carrier transport, and greater thermal stability [8]. These characteristics render FAPbI_3_ a highly promising candidate for photovoltaic applications.

However, the conventional synthesis of FAPbI_3_ typically utilizes low-temperature solution-based methods, a process that has been observed to result in the introduction of defects at the film surface and grain boundaries [9]. Moreover, FAPbI3 could not maintain stability at elevated temperatures. Prolonged exposure to high temperatures would trigger phase transitions and introduce defects into the crystal structure. Induced defects have been shown to promote non-radiative recombination and reduce carrier lifetime, ultimately compromising device performance [10].

To passivate defects surface states in perovskites, interest has turned to the use of organic molecular additives with functional groups. In recent years, multifunctional organic additives have demonstrated remarkable effectiveness in defect passivation and interfacial optimization in PSCs [11]. Zhu et al. [12] introduced 5-aminothiazole hydrochloride (5ATCl), a molecule featuring both electron-donating and electron-accepting functional groups, which effectively passivates defects and facilitates energy level alignment, resulting in PCEs of 26.38% and 24.54% for rigid and flexible PSCs, respectively, along with significantly enhanced device stability and scalability. Chen et al. [13] incorporated a thermally polymerizable bifunctional additive, N-(3-(dimethylamino)propyl)methacrylamide (DPM), into wide-bandgap perovskite films, enabling the formation of an in situ polymer network that suppresses ion migration and achieves a PCE of 18.19%, as well as 25.06% in tandem architectures. Mo et al. [14] developed a versatile additive, 4-amino-5-bromonicotinic acid (ABrNA), applicable to various perovskite compositions. This additive improves crystallinity and defect passivation, boosting the PCE of CsFAMA-based and MA-free devices to 25.0% and 23.0%, respectively, while exhibiting excellent stability and universality across multiple device architectures. Additionally, Li et al. [15] proposed aminomethylphosphonic acid (AMPA) as a multifunctional small-molecule additive that simultaneously modulates SnO_2_, the perovskite absorber, and their interface, enhancing device performance from 19.91% to 24.22% and delivering outstanding operational stability.

The molecules H_4_N_2_O_2_S, CH_8_N_4_O_3_S, and C_2_H_12_N_6_O_4_S are notable examples of sulfonamide (–SO_2_–NH_2_) functional group representation. This group is characterized by excellent chemical stability, and non-toxicity, and strong electron-withdrawing ability to form strong interactions with various surfaces. Liu et al. [16] developed a series of π-conjugated materials containing sulfonamide groups, which were utilized as binders for organic electrodes. The two-dimensional structures demonstrated a substantial enhancement in specific capacity and cycling stability, thereby demonstrating a notable enhancement in overall material performance and a robust application potential. Zhang et al. [17] investigated the manner in which disilicate forms hydrogen bonds with g-C3N4 and sulfonamide molecules, thereby significantly enhancing the photodegradation efficiency of SMZ. Notably, this group exhibits remarkable stability under elevated temperatures while concurrently functioning as both a hydrogen bond donor and acceptor [18]. This dual role contributes to the formation of robust hydrogen-bonding networks, which significantly enhance the overall stability of the materials. Among the identified groups, H_4_N_2_O_2_S and CH_8_N_4_O_3_S exhibit stable molecular structures and have the capacity to form hydrogen bonds, ionic bonds, or coordination interactions, rendering them widely applicable in molecular recognition and adsorption systems. C_2_H_12_N_6_O_4_S is notable for its exceptional chemical stability and ion-exchange capacity, and it can modulate materials’ structures and properties through intermolecular interactions, thereby enhancing the electrical conductivity of composite materials [19].

Inspired by the above-mentioned demonstrations of sulfonamide (–SO_2_–NH_2_) functional group, this work chose H_4_N_2_O_2_S, CH_8_N_4_O_3_S, and C_2_H_12_N_6_O_4_S as the additives for FAPbI_3_ perovskites to reveal the possibility for using the sulfonamide moiety-containing molecules to enhance the photoelectric properties of perovskites. Herein, DFT calculations were performed to investigate the adsorption behavior of selected sulfonamide-containing molecules on the FAPbI_3_(001) surface. The calculations focused on the adsorption energies, preferred adsorption sites, and the impact on the electronic structure of the perovskite surface. DOS and charge density difference analyses were performed to gain insight into electronic structure modifications upon molecular adsorption. Furthermore, the absorption spectra and imaginary part of the dielectric function were calculated to explore the optoelectronic properties of the adsorption systems. The aim was to elucidate the underlying mechanisms by which these sulfonamide-containing molecules interact with the perovskite surface and potentially enhance its photoelectric properties. By understanding these interactions, we hope to pave the way for the development of novel perovskite-based optoelectronic devices with improved performance characteristics.

## 2. Models and Computational Methods

**Figure 1:**
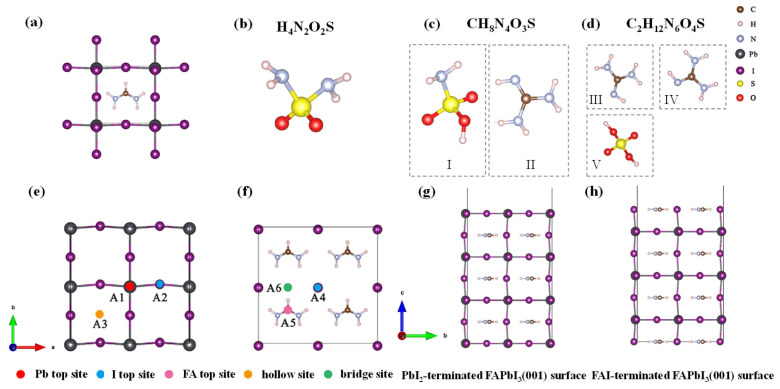
Crystal structure of (**a**) α-FAPbI_3_ unit cell, molecular structures of (**b**) H_4_N_2_O_2_S, (**c**) CH_8_N_4_O_3_S, and (**d**) C_2_H_12_N_6_O_4_S, adsorption sites of (**e**) PbI_2_-terminated and (**f**) FAI-terminated FAPbI_3_(001) surface, and surface structures of (**g**) PbI_2_-terminated and (**h**) FAI-terminated FAPbI_3_(001) surface.

First-principles calculations were performed based on the density functional theory (DFT) [20]. These calculations were carried out using the Vienna Ab initio Simulation Package (VASP) (Version 6.4.1) [21]. The exchange–correlation energy was treated within the framework of the generalized gradient approximation (GGA) using the Perdew–Burke–Ernzerhof (PBE) functional [22]. The interactions between electrons and ionic cores were described using the projector augmented-wave (PAW) method [23]. A plane-wave cutoff energy of 500 eV was employed [24]. The convergence criterion for atomic forces was set to 0.02 eV/Å [25], and the electronic self-consistent field (SCF) energy convergence threshold was set to 10^−5^ eV [26]. Structural optimizations of the FAPbI_3_ unit cell, as well as the H_4_N_2_O_2_S, CH_8_N_4_O_3_S, and C_2_H_12_N_6_O_4_S molecules were performed with a k-point sampling of 8 × 8 × 8 in the Brillouin zone (BZ). Our calculations using PBE functional yielded a band gap of 1.4743 eV for FAPbI_3_, which is in excellent agreement with the experimental value of approximately 1.48 eV. Since PBE underestimates the band gap, while the heavy metal element Pb has a spin-orbit coupling (SOC) effect that overestimates the band gap; hence, the two effects cancel each other out exactly. FAPbI_3_ features two commonly observed surface terminations: PbI_2_ and FAI [27]. Thus, the present study modeled these two types of surfaces, namely, the PbI_2_-terminated (Figure 1g) and FAI-terminated (Figure 1h) FAPbI_3_(001) surface. A 2 × 2 × 2 slab consisting of seven atomic layers was constructed to represent the surface, and a vacuum layer of 15 Å was introduced along a vertical direction to eliminate interactions between periodic images [28]. A k-point mesh of 2 × 2 × 1 was utilized for BZ sampling. During the structural optimization, the bottom four layers were fixed while the top three layers were allowed to relax. Subsequently, adsorption models were established by placing H_4_N_2_O_2_S, CH_8_N_4_O_3_S, and C_2_H_12_N_6_O_4_S molecules on both PbI_2_ and FAI-terminated FAPbI_3_(001) surface. The models are denoted as H_4_N_2_O_2_S (CH_8_N_4_O_3_S, C_2_H_12_N_6_O_4_S)/PbI_2_-surface and H_4_N_2_O_2_S (CH_8_N_4_O_3_S, C_2_H_12_N_6_O_4_S)/FAI-surface, respectively. The considered adsorption sites include the following: on top of a Pb atom (A1), on top of an I atom (A2), and a hollow site (A3) of the PbI_2_-terminated surface (Figure 1e); and on top of an I atom (A4), on top of the FA molecule (A5), and a bridge site (A6) of the FAI-terminated surface (Figure 1f).

## 3. Results and Discussion

### 3.1. Structural Properties of FAPbI_3_

FAPbI_3_ perovskite is a typical organic–inorganic hybrid optoelectronic material that can form α-phase at elevated temperatures and a yellow hexagonal δ-phase at low temperatures [29]. The α-phase, distinguished by its enhanced structural symmetry and superior visible light absorption properties [30], is particularly well-suited for utilization as a light-absorbing layer in perovskite solar cells [31]. The calculated lattice constants of the α-FAPbI_3_ are a = 6.369 Å, b = 6.425 Å, and c = 6.402 Å. These values are in good agreement with previously reported theoretical values (a = 6.361 Å, b = 6.508 Å, c = 6.323 Å) [32]. Considering the impact of organic molecules on crystal symmetry, a comparison was made using averaged lattice constant (6.399 Å) with the experimental value (6.397 Å) [33], which yielded an error of 0.002, suggesting the reliability of the structural parameters employed. The bandgap value 1.474 eV derived from our calculations aligns well with the experimental value of 1.48 eV [34] confirming the accuracy of our computational approach. Furthermore, the FA^+^ cation adopts a planar configuration, with the terminal –NH_2_ group orienting its hydrogen atoms toward the surrounding I^−^ ions, exhibiting a degree of spatial affinity. The calculated results indicate that the distances between the H atoms of the FA⁺ cation and the surrounding I^−^ ions in a FAPbI_3_ unit cell are 3.52 Å, 3.99 Å, 3.97 Å, and 3.73 Å. These distances fall within the range of weak hydrogen bonding (3.2–4.0 Å), as defined by George A. Jeffrey in An Introduction to Hydrogen Bonding [35]. Consequently, this interaction can be classified as a typical weak hydrogen bond. Despite the relatively low interaction strength, it contributes to the ordered orientation of FA⁺ cations within the lattice, thereby enhancing the structural stability of the crystal to a certain extent.

### 3.2. Adsorption Properties of Different Molecules on the FAPbI_3_(001) Surface

**Figure 2:**
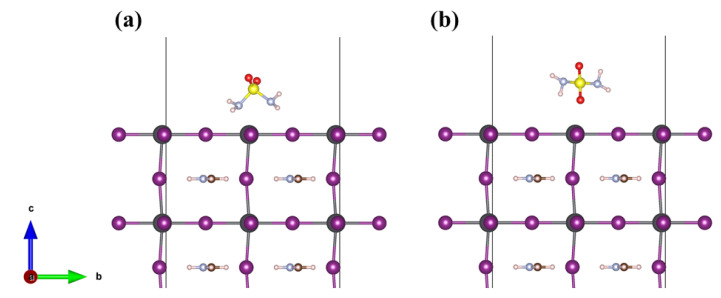
Different orientations of the H_4_N_2_O_2_S molecule on the FAPbI_3_(001) surface: (**a**) parallel orientation; (**b**) perpendicular orientation.

The adsorption behaviors of H_4_N_2_O_2_S, CH_8_N_4_O_3_S, and C_2_H_12_N_6_O_4_S molecules on the FAPbI_3_(001) surface were systematically investigated. Six representative adsorption sites (Figure 1e,f) were selected, and each molecule was initially placed on the FAPbI_3_(001) surface in both parallel and perpendicular orientations. For example, the configurations of H_4_N_2_O_2_S molecule were classified as two kinds, one is sulfonamide group oriented parallel to the FAPbI_3_(001) surface, as shown in Figure 2a and the other is the sulfonamide group oriented vertically to the FAPbI_3_(001) surface, as shown in Figure 2b. Subsequently, structural optimization was performed and the adsorption energies were calculated for each configuration to obtain the most energetically favorable configurations. This calculation enabled the quantification of the interaction strength between different molecules and the surface. The adsorption energy (E_ads_) of H_4_N_2_O_2_S, CH_8_N_4_O_3_S, and C_2_H_12_N_6_O_4_S molecules adsorbed on the FAPbI_3_(001) surface was calculated using the following equation:

(1) $$E_{\mathrm{ads}}{=E}_{\mathrm{total}}{-E}_{\mathrm{slab}}{-E}_{\mathrm{mol}}$$

where E_total_ is the total energy of whole system, i.e., the molecule adsorbed on FAPbI_3_(001) surface, E_slab_ is the energy of FAPbI_3_(001) surface, and E_mol_ is the energy of the molecule. The lower the adsorption energy, the more stable the adsorption.

**Figure 3:**
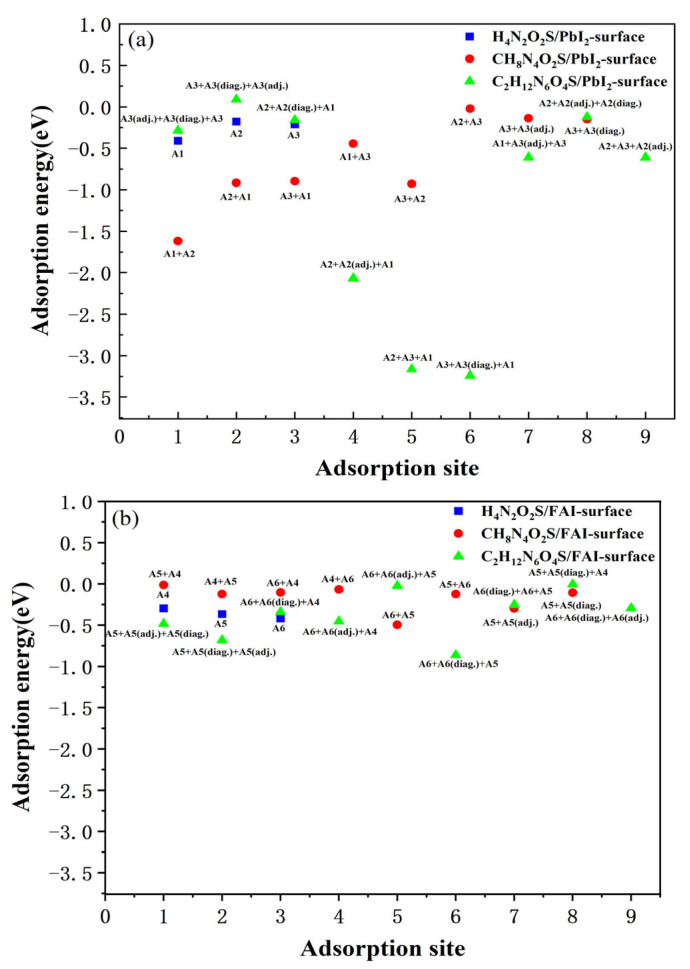
Adsorption energies (eV) of H_4_N_2_O_2_S, CH_8_N_4_O_3_S, and C_2_H_12_N_6_O_4_S molecules at different adsorption sites of (**a**) PbI_2_-terminated surface and (**b**) FAI-terminated surface.

The obtained adsorption energies for each adsorption configuration are listed in Table 1 and Figure 3. For the H_4_N_2_O_2_S molecule presenting just one segment, only three adsorption configurations were considered for each surface termination. The CH_8_N_4_O_3_S molecule is composed of two structural segments, designated as segment I and segment II, as illustrated in Figure 1c. For the PbI_2_-terminated FAPbI_3_(001) surface, there are five adsorption sites as illustrated in Figure 1e, named as A1, A2, A3, A3, A3(adj.) and A3(diag.). The terms diag. and adj. denote adsorption sites along the diagonal and adjacent lattice directions, respectively. Thereby, there are a total of eight different adsorption site combinations for molecules containing two segments, i.e., A1 + A2, A2 + A1, A3 + A1, A1 + A3, A3 + A2, A2 + A3, A3 + A3(adj.), and A3 + A3(diag.). Here, the order of the adsorption sites corresponds to the segment numbering. A1 + A2 means segment I of CH_8_N_4_O_3_S adsorbed on A1 and segment II adsorbed on A2, A2 + A1 means segment I of CH_8_N_4_O_3_S adsorbed on A2 and segment II adsorbed on A1. For C_2_H_12_N_6_O_4_S containing three segments, nine adsorption configurations were considered, the naming is also the same, each segment is corresponding to one adsorption site. The results indicate that all molecules exhibit negative adsorption energies on both surface terminations, suggesting stable adsorption on the FAPbI_3_(001) surface.

Further analysis reveals that the PbI_2_-terminated surface exhibits a notably stronger affinity towards CH_8_N_4_O_3_S and C_2_H_12_N_6_O_4_S, with the lowest adsorption energies reaching −1.618 eV and −3.242 eV, respectively. These values are significantly lower than those on the FAI-terminated surface, which are −0.495 eV and −0.862 eV. In contrast, the H_4_N_2_O_2_S molecule exhibits nearly equal adsorption energies on both surfaces (−0.410 eV and −0.419 eV), suggesting that its interaction with the FAPbI_3_ surface is less reliant on the specific surface termination. These structural features of CH_8_N_4_O_3_S and C_2_H_12_N_6_O_4_S bestow upon the molecules greater conformational flexibility and adaptability, allowing them to form multi-point interactions on the PbI_2_-terminated surface. The electron-loading sites in these molecules can effectively coordinate with the Pb atoms on the surface, and other interactions, such as hydrogen bonding, further enhancing their binding affinity. Consequently, the incorporation of carbon skeletons is imperative in enhancing the adsorption energy and structural stability of molecules on the PbI_2_-terminated surface.

The comparative analysis of multiple adsorption sites on the PbI_2_-terminated surface reveals that C_2_H_12_N_6_O_4_S exhibits the lowest adsorption energy among the three molecules. The most favorable configuration is located at the A3 + A3(diag.) + A1 sites, with the molecule adopting an overall nearly parallel orientation to the surface. In contrast, the optimal adsorption sites for H_4_N_2_O_2_S and CH_8_N_4_O_3_S were identified as A1 and A1 + A2, respectively. The superior adsorption performance of C_2_H_12_N_6_O_4_S is primarily attributed to the presence of multiple highly polar functional groups (–NH_2_ and –SO_3_H), which form stronger coordination interactions with surface Pb atoms. Furthermore, the molecular conformation of C_2_H_12_N_6_O_4_S facilitates cooperative adsorption at multiple active sites, thereby significantly enhancing interfacial binding strength. In comparison, CH_8_N_4_O_3_S exhibits moderate adsorption stability, while H_4_N_2_O_2_S demonstrates the weakest binding, indicating relatively low surface affinity. On the FAI-terminated surface, C_2_H_12_N_6_O_4_S molecule exhibits the strongest adsorption capability, with its most stable configuration located at the A6 + A6(diag.) + A5 adsorption sites. The molecule maintains a nearly parallel orientation with the surface, exhibiting remarkable conformational adaptability and interfacial binding capability. In contrast, the adsorption effect of H_4_N_2_O_2_S on this surface remains limited. In summary, H_4_N_2_O_2_S exhibits the simplest structure, lacking carbon skeleton and containing only one sulfonamide (–SO_2_–NH_2_) functional group. The reduced number of available adsorption sites and the exclusive nature of the interaction result in the primary interaction with the perovskite surface through local hydrogen bonding or weak coordination. This interaction leads to a comparatively weaker adsorption strength. In contrast, the CH_8_N_4_O_3_S and C_2_H_12_N_6_O_4_S molecules contain multiple carbon atoms that form more extended molecular frameworks and carry several (–SO_2_–NH_2_) functional groups. These molecules exhibit stronger surface coverage and multi-site coordination capabilities and strong potential for interfacial engineering applications.

**Figure 4:**
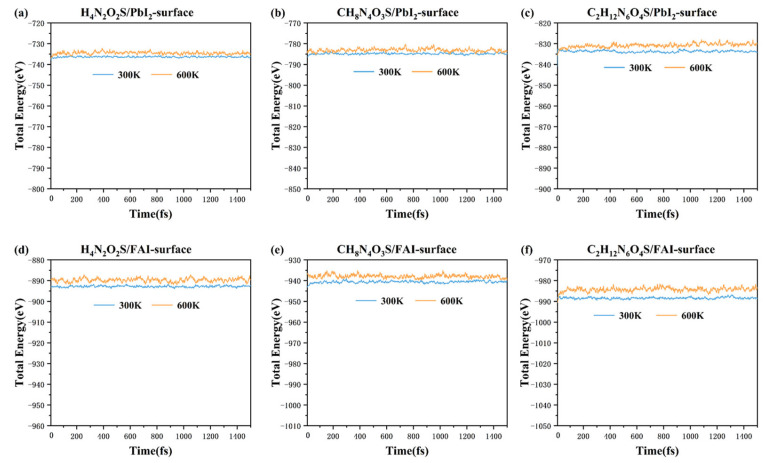
The stability tests of AIMD (NVT) calculations at 300 K and 600 K of (**a**) H_4_N_2_O_2_S, (**b**) H_8_N_4_O_3_S, and (**c**) C_2_H_12_N_6_O_4_S molecules adsorbed on the PbI_2_-terminated surface, and (**d**) H_4_N_2_O_2_S, (**e**) CH_8_N_4_O_3_S, and (**f**) C_2_H_12_N_6_O_4_S molecules adsorbed on FAI-terminated surface.

To further verify the adsorption stability of the three molecules on the FAPbI_3_(001) perovskite surface, ab initio molecular dynamics (AIMD) simulations were performed. The most favorable configurations of H_4_N_2_O_2_S, CH_8_N_4_O_3_S, and C_2_H_12_N_6_O_4_S on both PbI_2_- and FAI-terminated surfaces were simulated with canonical ensemble (NVT) at 300 K and 600 K for 1500 fs. As illustrated in Figure 4, the results indicated that the energy fluctuations across all systems were minimal at both temperatures, and there was an absence of substantial desorption or structural reconstruction of the adsorbed molecules, suggesting good thermal stability at elevated temperatures of purposed molecules adsorbed on the FAPbI_3_(001) surface.

### 3.3. Electronic Properties of Different Molecules on the FAPbI_3_(001) Surface

**Figure 5:**
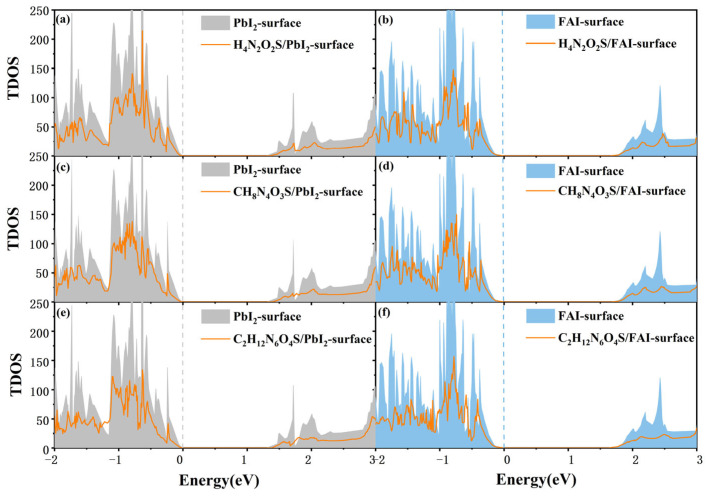
Total density of states (TDOS) of PbI_2_-terminated and FAI-terminated surface before and after adsorption of (**a**,**b**) H_4_N_2_O_2_S, (**c**,**d**) CH_8_N_4_O_3_S, and (**e**,**f**) C_2_H_12_N_6_O_4_S molecules. The gray and blue areas represent the TDOS before adsorption, and the orange line represents the TDOS after adsorption.

Subsequent analyses were conducted to evaluate the impact of molecular adsorption on the FAPbI_3_(001) surface by the most stable configurations. In order to gain deeper insight into the electronic structure modulation induced by the three molecules on the FAPbI_3_(001) surface, total density of states (TDOS) analyses were performed. As illustrated in Figure 5, the orange curves depict the TDOS of the systems following molecular adsorption, with the shaded gray regions denoting the reference TDOS of the pristine surface. The results indicate that molecular adsorption leads to varying degrees of electronic structure reconstruction at both the VBM and CBM. Specifically, Figure 5c,e demonstrate significant alterations in TDOS of CH_8_N_4_O_3_S and C_2_H_12_N_6_O_4_S on the PbI_2_-terminated surface, accompanied by discernible shifts in the electronic state distribution near the band edges in comparison to the unmodified surface. These observations suggest that molecular adsorption effectively modulates the distribution of electronic states, a phenomenon that may be attributed to enhanced overlap between molecular orbitals and surface states or increased orbital coupling effects. Such electronic reconstruction at the interface has been shown to be beneficial for tuning charge dynamics, potentially facilitating electron injection and improving device efficiency. On the FAI-terminated surface, the alterations in TDOS upon molecular adsorption are relatively insignificant; the TDOS profiles before and after adsorption remain largely analogous, suggesting a weak coupling between the molecules and FAI-terminated surface. A comparative analysis of the three systems reveals that both molecular complexity and surface termination type significantly influence the modulation of TDOS. The larger molecule, C_2_H_12_N_6_O_4_S, has been shown to induce more pronounced electronic state redistribution on the PbI_2_-terminated surface. This effect is likely due to the molecule’s multiple electron-donating and electron-withdrawing groups and its broader spatial coverage, which enhance interactions with surface states. In summary, the extent of electronic structure reconstruction correlates with the adsorption strength. These results offer valuable insights for achieving interfacial energy level alignment and optimizing charge transport in perovskite optoelectronic devices.

To further analyze the changes in interfacial electronic structure, the work function (ψ) was calculated, which reflects the intrinsic photoemission capability of the material. The calculation is based on the following formula:

ψ = E_vacuum_ − E_f_ (2)

**Figure 6:**
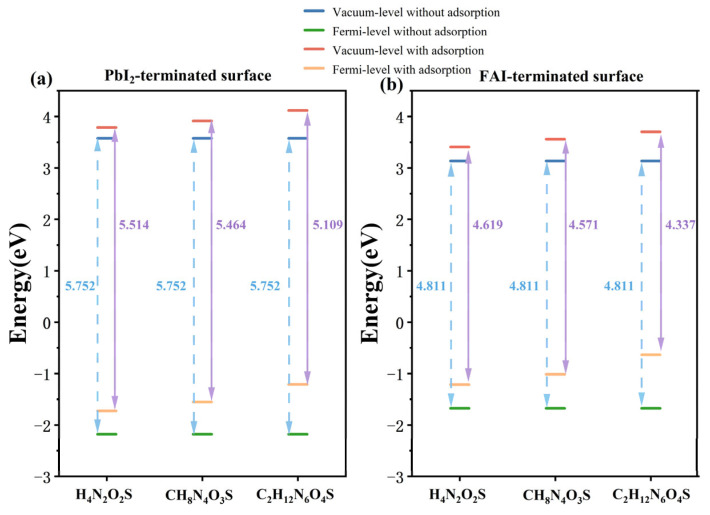
Fermi-level and vacuum-level of (**a**) PbI_2_-terminated surface and (**b**) FAI-terminated surface with and without molecular adsorption.

Here, E_vacuum_ and E_f_ represent the vacuum-level and the Fermi-level, respectively. As shown in Figure 6, the vacuum-level and Fermi-level of FAPbI_3_ surface with and without molecular adsorption were illustrated. The results indicated that on both PbI_2_-terminated and FAI-terminated surface, molecular adsorption results in a significant reduction in the work function, with both the vacuum-level and Fermi-level of all systems being up-shifted. The reduction in work function is due to the upward shift of the Fermi-level, which is more significant than that of the vacuum-level, consequently leading to a lower energy barrier for electron emission. This observation indicated the presence of robust electronic coupling between the molecules and the surface, thereby facilitating the alignment of interfacial energy-levels. Furthermore, the degree of reduction in work function after adsorption of different molecules follows: C_2_H_12_N_6_O_4_ > CH_8_N_4_O_3_S > H_4_N_2_O_2_S, these findings also confirmed our previous conclusion that molecules with expanded carbon frameworks and more –SO_2_–NH_2_ functional groups would be better for the surface modification.

**Figure 7:**
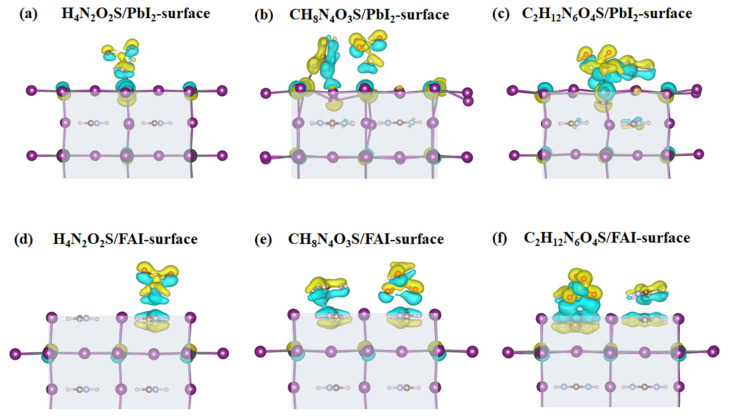
The plots of charge density difference (CDD) of (**a**) H_4_N_2_O_2_S, (**b**) CH_8_N_4_O_3_S, (**c**) C_2_H_12_N_6_O_4_S adsorbed on the PbI_2_-terminated surface, and (**d**) H_4_N_2_O_2_S, (**e**) CH_8_N_4_O_3_S, (**f**) C_2_H_12_N_6_O_4_S adsorbed on the FAI-terminated surface, where yellow and cyan regions correspond to charge accumulation and depletion zones, respectively, with an isosurface value of 0.001 electron bohr^−3^.

The charge transfer between the adsorbed molecule and substrate is the primary driving force behind the molecular adsorption on the FAPbI_3_(001) surface. To gain deeper insight into the interactions between H_4_N_2_O_2_S, CH_8_N_4_O_3_S, and C_2_H_12_N_6_O_4_S with the FAPbI_3_(001) surface, the CDD analysis was performed. The CDD is calculated using the following equation:

(3) $$\Delta \rho =\rho_{total}-\rho_{slab}-\rho_{mol}$$

where ρ_total_, ρ_slab_, and ρ_mol_ represent the charge density of the molecule adsorbed on the FAPbI_3_(001) surface, the FAPbI_3_(001) surface without molecule, and the isolated molecule (i.e., H_4_N_2_O_2_S, CH_8_N_4_O_3_S, C_2_H_12_N_6_O_4_S), respectively. CDD mapping provides an intuitive visualization of charge redistribution during the adsorption, offering valuable insights into the electronic coupling characteristics between the molecules and the perovskite surface. In Figure 7, the shaded region shows the FAPbI_3_(001) substrate, while the yellow and cyan regions correspond to charge accumulation and depletion zones, respectively. As illustrated in Figure 7a–c, all three molecules demonstrate substantial charge redistribution at the interface on the PbI_2_-terminated surface, suggesting robust orbital overlap and significant electronic interaction with surface states. As illustrated in Figure 7a, charge redistribution is predominantly localized between the O and S containing groups of the molecule and the surface Pb atoms, suggesting the formation of Pb–O and Pb–S coordination bonds. In Figure 7c, the presence of multiple regions of charge accumulation, particularly surrounding the –OH and –NH_2_ groups, suggests that the adsorption stability may stem from a combination of hydrogen bonding and weak coordination interactions. As illustrated in Figure 7e, the larger C_2_H_12_N_6_O_4_S molecule, with its multiple functional groups (–SO_2_–NH_2_), exhibits continuous charge accumulation zones surrounding multiple N, O, and S atoms, indicative of a multi-site coordination adsorption mode. This molecule is likely to form a variety of coordination bonds simultaneously (e.g., Pb–N, Pb–O, and Pb–S), demonstrating strong interfacial synergistic interaction capability. In contrast, for the FAI-terminated surface (Figure 7d–f), the observed charge redistribution and bonding are comparatively weaker and more localized. The interactions between the molecules and the surface are likely dominated by hydrogen bonding and electrostatic adsorption, with limited directional coordination features. In comparison to the PbI_2_-terminated surface, the FAI-terminated surface manifests a more inert interfacial character towards H_4_N_2_O_2_S, CH_8_N_4_O_3_S, C_2_H_12_N_6_O_4_S. These findings indicate that the adsorption behavior is governed by a synergistic interplay among surface termination type, charge redistribution extent, and molecular spatial configuration.

To quantitatively assess the interfacial charge transfer, Bader charge analysis was performed. The results (listed in Table 2) indicated that all three molecules induced significant charge transfer upon adsorption on the FAPbI_3_(001) surface, with the effect being most pronounced on the PbI_2_-terminated surface. Specifically, Pb atoms on the PbI_2_-terminated surface gain more electrons (+0.93 to +0.98 e^−^), significantly higher than those on the FAI-terminated surface (+0.84 to +0.91 e^−^), indicating stronger electronic coupling at the interface. Consequently, the O, N, and S atoms in the molecules experience a greater loss of electrons on the PbI_2_-terminated surface, particularly the S atom in C_2_H_12_N_6_O_4_S, which exhibits a charge loss of −0.64 e^−^ compared to −0.59 e^−^ on the FAI-terminated surface. This is consistent with the CDD plots (as shown in Figure 7) that electrons migrate from the surface Pb atoms and I^−^ ions to the more electronegative O and S atoms within the adsorbed molecules, resulting in significant interfacial charge redistribution.

### 3.4. Optical Properties of Different Molecules Adsorbed on the FAPbI_3_(001) Surface

The real (ε_1_) and imaginary (ε_2_) parts of complex dielectric function along the XX and YY directions were calculated using the PBE approximation for the FAPbI_3_(001) systems with H_4_N_2_O_2_S, CH_8_N_4_O_3_S, and C_2_H_12_N_6_O_4_S molecules adsorbed on both PbI_2_- and FAI-terminated surfaces. To obtain the influence of these molecules on optical absorption properties of the FAPbI_3_(001) surface, the corresponding optical absorption coefficients were also computed. The absorption coefficient, I(ω), is calculated as follows:

(4) $$I(\omega )=\sqrt{2}\omega {\left[\sqrt{\varepsilon_{1}{\left(\omega \right)}^{2}+\varepsilon_{2}{\left(\omega \right)}^{2}}-\varepsilon_{1}(\omega )\right]}^{1/2}$$

**Figure 8:**
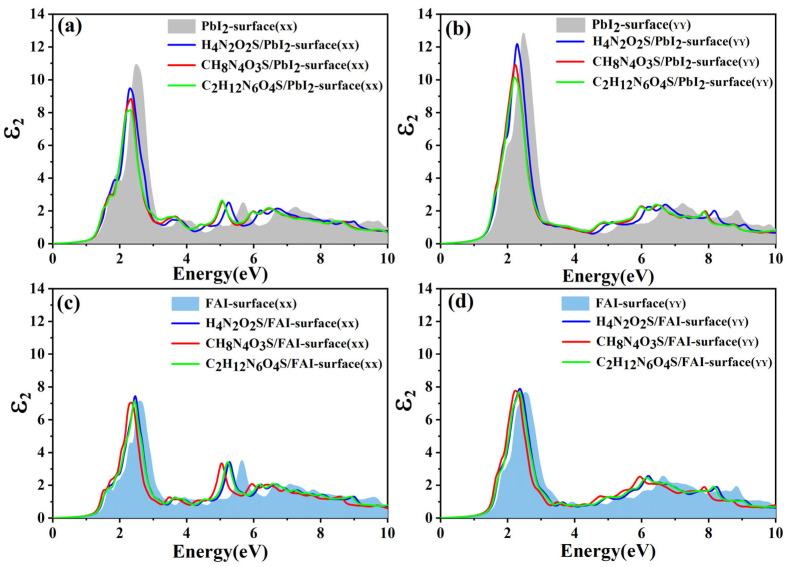
Imaginary part (ε_2_) of the dielectric function of H_4_N_2_O_2_S, CH_8_N_4_O_3_S, C_2_H_12_N_6_O_4_S adsorbed on the PbI_2_-terminated surface along (**a**) XX and (**b**) YY direction, and adsorbed on the FAI-terminated surface along (**c**) XX and (**d**) YY direction. The gray and blue shading represent the clean PbI_2_-terminated and FAI-terminated surface, respectively.

The real part (ε_1_) of the dielectric function is representative of the real component of the complex dielectric constant [36], while ε_2_ represents the imaginary component, and ω corresponds to the light frequency. As demonstrated in Figure 8, the variations in the imaginary part of the dielectric function (ε_2_) along the XX and YY directions within the 0–10 eV energy range are presented for the FAPbI_3_(001) systems with H_4_N_2_O_2_S, CH_8_N_4_O_3_S, and C_2_H_12_N_6_O_4_S molecules adsorbed. Figure 8a,b correspond to the PbI_2_-terminated surface, while Figure 8c,d represent the FAI-terminated surface. The shaded regions in these figures denote the ε_2_ profiles of the respective clean surfaces. A comparison of pristine surfaces with all adsorption systems reveals a pronounced enhancement in ε_2_ values in the low-energy region (0–3 eV), accompanied by a shift in the main peak towards higher energies. This observation indicates that molecular adsorption leads to the formation of novel electronic states. This phenomenon results in a decrease in transition energy and an increase in optical transition intensity near the band edge. Furthermore, the disparity between the XX and YY directional responses points to enhanced optical anisotropy, which may stem from an asymmetric interfacial charge distribution resulting from molecular adsorption. It is noteworthy that all adsorption configurations exhibit a shift towards higher energy. A comprehensive analysis reveals that the ε_2_ values on the PbI_2_-terminated surface exceed those on the FAI-terminated surface. This observation suggests the presence of more robust electronic interactions between the adsorbed molecules and PbI_2_-terminated surface. These interactions are likely attributable to charge redistribution effects associated with unsaturated Pb sites. Furthermore, molecular adsorption results in a broader spectral absorption range, and a decrease in peak intensity becomes more pronounced with increasing molecular complexity. In summary, molecular adsorption significantly modulates the optical response of the FAPbI_3_(001) surface, primarily by introducing interfacial states and altering electronic transition probabilities, thereby enabling effective tuning of its optical properties.

**Figure 9:**
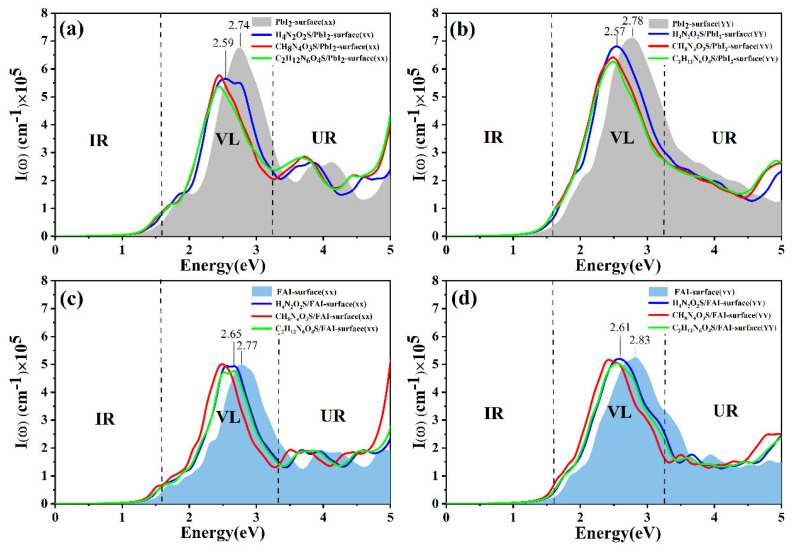
Absorption spectra of H_4_N_2_O_2_S, CH_8_N_4_O_3_S, C_2_H_12_N_6_O_4_S adsorbed on the PbI_2_-terminated surface along (**a**) XX and (**b**) YY direction, and adsorbed on the FAI-terminated surface along (**c**) XX and (**d**) YY direction. The gray and blue shading represent the clean PbI_2_-terminated and FAI-terminated surface, respectively.

As illustrated in Figure 9, the optical absorption coefficient I (ω) is presented as a function of photon energy in the range of 0–5 eV for the FAPbI_3_(001) surfaces with H_4_N_2_O_2_S. CH_4_N_2_O_2_S, and C_2_H_12_N_6_O_4_S molecules adsorbed along the XX and YY directions. Figure 9a,b correspond to the PbI_2_-terminated surface, while Figure 9c,d represent the FAI-terminated surface. The shaded areas in the figures denote the absorption profiles of the respective clean surfaces. As demonstrated in the results of the dielectric function analysis presented in Figure 8, all molecular adsorption systems manifest a discernible red shift in the absorption edge, indicative of augmented light absorption capacity in the visible (VL) and near-infrared (IR) regions. This enhancement has been demonstrated to be advantageous in enhancing the light-harvesting efficiency of perovskite materials under conditions of low luminosity. The red shift is primarily attributed to band structure reconstruction and the introduction of new electronic states induced by molecular adsorption. These phenomena lower the excitation threshold and enhance the system’s responsiveness to VL and IR light. Concurrently, the red shift is indicative of robust interfacial coupling between the molecules and the perovskite surface, predominantly via multi-point coordination. This phenomenon enhances orbital coupling and delocalization of electronic states, consequently modulating the interfacial energy level structure. A notable observation is the substantial variation in the absorption intensity exhibited by the different molecules. H_4_N_2_O_2_S demonstrated the highest absorption coefficient following adsorption, while C_2_H_12_N_6_O_4_S exhibited the lowest. This observation highlights the sensitivity of interfacial optical response to molecular structure. As illustrated in Figure 9a,b, the absorption edge undergoes a shift from approximately 2.74 eV to 2.57 eV on the PbI_2_-terminated surface. This shift is accompanied by a significant enhancement in the 1.6–3.2 eV visible light range, particularly along the XX polarization direction. Conversely, on the FAI-terminated surface, the absorption edge shifts from approximately 2.77 eV to 2.65 eV, exhibiting a more moderate change in absorption. This suggests that the PbI_2_-terminated surface is more conducive to functioning as an optically tunable interface. Subsequent analysis indicates that, while the absorption intensity undergoes a slight decrease in certain directions, the overall spectral coverage remains stable, devoid of significant blind spots. This property is advantageous for preserving broad-spectrum device functionality. Despite the absence of AM1.5 spectral overlap analysis and device-level PCE simulations in this study, the absorption spectra, density of states, and charge distribution results have already demonstrated the potential of molecular modification in enhancing visible light absorption and tuning interfacial properties. In summary, molecular adsorption has been demonstrated to enhance the visible light response of the FAPbI_3_(001) perovskite surface. Furthermore, this phenomenon provides a theoretical foundation for the design of high-efficiency perovskite optoelectronic devices based on interfacial engineering.

## 4. Conclusions

In this study, first-principles calculations were conducted to systematically investigate the adsorption behavior of sulfonamide-containing molecules—H_4_N_2_O_2_S, CH_8_N_4_O_3_S, and C_2_H_12_N_6_O_4_S—on the FAPbI_3_(001) surface. The results indicated that all three molecules demonstrate stable adsorption capabilities on the FAPbI_3_(001) surface, with C_2_H_12_N_6_O_4_S exhibiting the most optimal interfacial binding stability due to its electron-donating and electron-withdrawing groups, extended carbon frameworks, and functional groups. AIMD simulations indicated that all adsorption configurations exhibit adequate thermal stability at both 300 K and 600 K. Electronic structure analysis revealed that molecular adsorption significantly reconstructed the DOS of the PbI_2_-terminated surface, causing a shift in band-edge states and enhancing energy-level coupling between molecular orbitals and surface states. Conversely, the FAI-terminated surface exhibited a diminished interaction. Work function analysis indicated that molecular adsorption on the surface significantly reduces the work function, particularly in the case of C_2_H_12_N_6_O_4_S. CDD analysis further indicated that the molecules formed multiple coordination bonds (such as Pb–O, Pb–S, and Pb–N) with uncoordinated Pb atoms, assisted by –SO_2_–NH_2_ groups. Bader charge analysis revealed that charge transfer is more pronounced on the PbI_2_-terminated surface, with Pb atoms gaining electrons and the O, N, and S atoms of the molecules showing significant electron loss. Furthermore, calculations of optical properties revealed that all adsorption systems exhibited a distinct red-shift in the absorption edge along both the XX and YY directions, accompanied by enhanced absorption intensity and broadened spectral range.

The collective findings indicated that the sulfonamide moiety-containing molecules examined, particularly C_2_H_12_N_6_O_4_S with extended carbon frameworks and functional groups, contribute significantly to the stabilization of the perovskite interface, the optimization of charge transport pathways, and the enhancement of light-harvesting performance. This theoretical work confirmed the potential of sulfonamide-based molecules as effective interfacial additives and provided mechanistic insights into their interfacial regulatory roles in perovskite materials, offering valuable guidance for the rational design of high-efficiency and stable perovskite optoelectronic devices. In subsequent studies, the incorporation of solvent models and additional molecular dynamics simulations will be undertaken to achieve a more precise representation of the experimental conditions.

## Figures and Tables

**Table 1:** Adsorption energies of different molecules at different adsorption sites of PbI_2_-terminated and FAI-terminated FAPbI_3_(001) surface. The terms diag. and adj. denote adsorption sites along the diagonal and adjacent lattice directions, respectively. The naming of adsorption sites follows the sequence of segmented structural units illustrated in Figure 1c,d.

	PbI_2_-Terminated Surface		FAI-Terminated Surface	
Molecule	Adsorption Sites	Adsorption Energy (eV)	Adsorption Sites	Adsorption Energy (eV)
H_4_N_2_O_2_S	A1	−0.41	A4	−0.296
	A2	−0.178	A5	−0.367
	A3	−0.211	A6	−0.419
CH_8_N_4_O_3_S	A1 + A2	−1.618	A5 + A4	−0.015
	A2 + A1	−0.917	A4 + A5	−0.125
	A3 + A1	−0.896	A6 + A4	−0.105
	A1 + A3	−0.444	A4 + A6	−0.069
	A3 + A2	−0.928	A6 + A5	−0.495
	A2 + A3	−0.021	A5 + A6	−0.124
	A3 + A3(adj.)	−0.139	A5 + A5(adj.)	−0.298
	A3 + A3(diag.)	−0.15	A5 + A5(diag.)	−0.107
C_2_H_12_N_6_O_4_S	A3(adj.) + A3(diag.) + A3	0.285	A5 + A5(adj.) + A5(diag.)	−0.482
	A3 + A3(diag.) + A3(adj.)	0.092	A5 + A5(diag.) + A5(adj.)	−0.682
	A2 + A2(diag.) + A1	−0.158	A6 + A6(diag.) + A4	−0.341
	A2 + A2(adj.) + A1	−2.068	A6 + A6(adj.) + A4	−0.454
	A2 + A3 + A1	−3.163	A6 + A6(adj.) + A5	−0.023
	A3 + A3(diag.) + A1	−3.242	A6 + A6(diag.) + A5	−0.862
	A1 + A3(adj.) + A3	−0.608	A6(diag.) + A6 + A5	−0.253
	A2 + A2(adj.) + A2(diag.)	−0.122	A5 + A5(diag.) + A4	−0.007
	A2 + A3 + A2(adj.)	−0.609	A6 + A6(diag.) + A6(adj.)	−0.293

**Table 2:** Bader charge analysis of the adsorption systems (“+” indicates electron gain; “−” indicates electron loss; “surface” refers to atoms on the FAPbI_3_(001) surface).

	PbI_2_-Terminated Surface		FAI-Terminated Surface	
Molecule	Atom	Bader Charge (e)	Atom	Bader Charge (e)
H_4_N_2_O_2_S	Pb(surface)	+0.93	Pb(surface)	+0.84
	O	−0.25	O	−0.25
	N	−0.36	N	−0.31
	N(surface)	−0.10	N(surface)	−0.11
	S	−0.12	S	−0.13
	I(surface)	−0.02	I(surface)	−0.07
CH_8_N_4_O_3_S	Pb(surface)	+0.97	Pb(surface)	+0.86
	O	−0.27	O	−0.27
	N	−0.25	N	−0.22
	N(surface)	−0.11	N(surface)	−0.12
	S	−0.49	S	−0.4
	I(surface)	−0.04	I(surface)	−0.08
C_2_H_12_N_6_O_4_S	Pb(surface)	+0.98	Pb(surface)	+0.91
	O	−0.33	O	−0.31
	N	−0.36	N	−0.35
	N(surface)	−0.12	N(surface)	−0.14
	S	−0.64	S	−0.59
	I(surface)	−0.05	I(surface)	−0.09

## Data Availability

Data will be made available on request.
